# The Apathy Evaluation Scale (AES-C): Psychometric Properties and Invariance of Italian Version in Mild Cognitive Impairment and Alzheimer’s Disease

**DOI:** 10.3390/ijerph18189597

**Published:** 2021-09-12

**Authors:** Giovanna Furneri, Silvia Platania, Alessandra Privitera, Federica Martelli, Rossana Smeriglio, Grazia Razza, Tiziana Maci, Sabrina Castellano, Filippo Drago, Mario Santagati, Pasquale Caponnetto, Filippo Caraci, Santo Di Nuovo

**Affiliations:** 1 Department of Educational Sciences, University of Catania, 95124 Catania, Italy; giofurneri@gmail.com (G.F.); splatani@unict.it (S.P.); alessandra.privitera0@gmail.com (A.P.); federica.martelli95@tiscali.it (F.M.); Sabrina.castellano@unict.it (S.C.); 2 Department of Biomedical and Biotechnological Sciences, University of Catania, 95124 Catania, Italy; f.drago@unict.it; 3 Oasi Research Institute—IRCCS, 94018 Troina, Italy; rossanasmeriglio@gmail.com (R.S.); fcaraci@unict.it (F.C.); 4 ASP3 Catania, Department of Mental Health, Alzheimer Psychogeriatric Center, 95127 Catania, Italy; grazia.razza@aspct.it (G.R.); tiziana.maci@aspct.it (T.M.); mario.santagati@aspct.it (M.S.); 5 Center of Excellence for the Acceleration of Harm Reduction (COEHAR), University of Catania, 95124 Catania, Italy; 6 Department of Drug and Health Sciences, University of Catania, 95124 Catania, Italy

**Keywords:** apathy, AES-C, validation, reliability, psychometric properties, Alzheimer, MCI

## Abstract

Apathy is a neuropsychiatric symptom observed in different neurological and psychiatric disorders. Although apathy is considered a symptom, it has been recently reconsidered as a syndrome characterised by three dimensions: cognitive symptoms, affective symptoms and behavioural symptoms. Recent studies have shown that apathy can be considered as a prodromal symptom of Alzheimer’s disease (AD), but also an indicator of the transition from mild cognitive impairment to AD. According to this scenario, an early detection of apathy in subjects with Mild Cognitive Impairment (MCI) and Mild AD can be a valid psychometric strategy to improve an early diagnosis and promote a prompt intervention. The Apathy Evaluation Scale is a validated tool composed of 18 items that assess and quantify emotional, behavioural and cognitive aspects of apathy. The aim of this study is to assess the specific reliability and validity of the Italian version of the Apathy Evaluation Scale—Clinician Version (AES-C) to detect apathy both in amnestic MCI and mild AD patients. In the present paper, we therefore examined the psychometric properties and the invariance of the Italian Version of the AES-C conducted on a sample composed of an experimental group of amnestic MCI and AD patients (N = 107) and a control group (N = 107) constituted by Age- and Sex-matched healthy controls. Results confirm the goodness of the scale. Confirmatory factory analysis confirmed that the AES-C Italian Version presents the same stability of one second-order factor and three first-order factors identified in the original version, and all items are predicted by a single general factor. Moreover, the scale was found to be invariant across both populations. Moreover, reliability and discriminant analysis showed good values. We found in the experimental group a negative correlation between the AES-C and Frontal Assessment Battery (FAB) (rs = −0.21, *p* < 0.001) and Mini Mental State Examination (MMSE) (rs = −0.04, *p* < 0.001), while a positive correlation was found between the AES-C and Hamilton psychiatric Rating scale for Depression (HAM-D) scores (rs = 0.58, *p* < 0.001) Overall, our data demonstrated the validity of the Italian version of the AES-C for the assessment of apathy both in MCI and in AD patients.

## 1. Introduction

Apathy is a neuropsychiatric symptom (NPS) observed in different neurological and psychiatric disorders [1,2]. Etymologically, its name comes from the Greek word ἀπάθεια (apatheia), meaning lack (a-) of emotions (-pathos), and some of our Greek and Roman ancestors, in the frame of stoic philosophy, considered it as a virtue. So far from this perspective, the current scientific point of view regards apathy as a prodromal symptom in different diseases such as multiple sclerosis, amyotrophic lateral sclerosis, psychosis, traumatic brain injury and all type of dementia, especially in both Alzheimer’s disease (AD) and vascular dementia (VaD) [3].

Apathy’s main feature is a deficit in motivation, which is displayed through reduced interest, emotions and goal-directed behaviours. Differently from depression, apathy is characterised by emotional neutrality rather than negative emotions [4]. It is a multidimensional construct that includes the emotional and social sphere and is characterised by three dimensions: cognitive symptoms, affective symptoms and behavioural symptoms [5]. Apathy has often been regarded as a symptom of other syndromes (e.g., depression or dementia) [6], but it can itself be defined as a syndrome characterised by decreased motivation in which the lack of motivation is not attributable to a decreased level of consciousness, emotional distress or cognitive deficits [7].

In the last two decades, the interest about the association between apathy and dementia has increased due to the predictive role of apathy on dementia. AD is a progressive and irreversible neurodegenerative disease that usually affects people over the age of 65 [8] and is the most common form of dementia [9,10]. The onset of AD begins many years before the actual diagnosis [11] and is characterised by a preclinical and prodromal phase that can begin as early as middle age [12] AD is characterised by its increasing neuropsychological impairment with a progressive decline in memory, executive functions, language and visuospatial skills [13]. In almost all patients, the cognitive and functional decline of the disease is accompanied by behavioural and psychological symptoms (BPSD) [14] such as apathy, agitation, disappointment, irritability, anxiety, disinhibition and hallucinations [13], i.e., symptoms of disturbed perception, thought content, mood, and behaviour [15] which significantly reduce the quality of life of patients and caregivers [16,17]. According to this scenario, it is now widely demonstrated that apathy can act as a risk factor which can promote the conversion from Mild Cognitive Impairment (MCI) to dementia.

Chilovi et al. [18] classified MCI patients in four subgroups, namely (1) MCI normal, (2) MCI depressed, (3) MCI depressed-apathetic and (4) MCI apathetic. They found the highest percentage of conversion to dementia among the apathetic group (60%), followed by MCI normal (24%), MCI depressed-apathetic (19%) and MCI depressed (7.9%). Similarly, in a sample of 1713 MCI patients, it was observed that apathetic subjects have a greater risk of developing dementia than those with depression only [19]. More recently, with a cohort of 2137 MCI patients, Roberto et al. [20] found fairly the same results. They divided the sample in four classes based on the predominant NPS symptoms: (1) irritability, (2) apathy, (3) anxiety/depression, (4) asymptomatic. While irritability and apathy were predictors of dementia, anxiety/depression did not constitute a risk factor. These results are confirmed by a recent systematic review [21] that investigated the impact of apathy, depression and anxiety on the progression from MCI to AD. The authors underlined that for MCI patients, apathy is a ‘more important indicator’ among the two other neuropsychiatric symptoms in the evolution to dementia.

Thus, an early assessment and identification of apathy are extremely useful for clinicians to understand the possible evolution from MCI to Mild AD better and therefore design tailored prevention and treatment programs.

Different tools are available in the literature as psychometric tools for assessing apathy. The Neuropsychiatric Inventory (NPI), for example, developed by Cumming [22,23], is a multi-dimensional instrument to assess neurobehavioural disorders in dementia. In particular, it presents a specific eight-item subscale to measure apathy: higher scores indicate a more severe presence of apathy [22,23]. Robert et al. [24] developed the Apathy Inventory (AI) on the basis of Marin and colleagues’ diagnostic criteria of apathy [6,7], but also Starkstein and colleagues [25] and Lueken et al. [26] settled two different and modified short versions of the Marin instrument. Strauss and Sperry [27] developed the Dementia Apathy Interview and Rating (DAIR) which is an informant-based unidimensional scale that measures changes in engagement, motivation and emotional response in MCI patients. The Lille Apathy Rating Scale (LARS) is based on a structured interview. It includes 33 items, divided into nine domains. Responses are scored on a dichotomous scale. The scale was validated with a sample of 159 patients with probable Parkinson’s disease and 58 healthy controls [28]. In subsequent studies, LARS was also administered to other disease populations, such as AD and MCI patients [29].

Radakovic and Abrahams [30] created a multi-dimensional scale based on Levy and Dubois’ [31] apathetic subtypes. The Dimensional Apathy Scale (DAS) is composed of 24 items and 3 subscales—Executive, Emotional and Behavioural/Cognitive Initiation. DAS is suitable for application in different pathologies.

In the present work, we performed the validation and psychometric analysis of the Italian Version of the Apathy Evaluation Scale—Clinician Version (AES-C) on MCI and Mild AD patients starting from the evidence that AES is based on a definition of apathy that is closer to a syndrome in and of itself rather than as a symptom of other syndromes. The Apathy Evaluation Scale (AES) was developed by Marin and colleagues [32] and was conceived and built based on the definition of apathy according to Marin: syndrome of loss of motivation as reflected by acquired changes in affect (mood), behaviour and cognition [32]. The AES is a four-point Likert-Scale measure, composed of 18 items that assess and quantify emotional, behavioural and cognitive aspects of apathy. AES presents three available administration forms of the scale: self-report (AES-S), informant report (AES-I) and clinician interview (AES-C). Only in the AES-C is a semi-structured open-ended interview present: it helps the clinician to collect information from patients about their typical day and hobbies and interests; this interview allows the clinician to investigate the subject’s degree of motivation and direct him in providing his own rating of the individual’s level of apathy on each item. Every item is rated on a four-point response scale (0 = not at all true/characteristic to 3 = very much true/characteristic). Higher scores indicate more severe apathy [32]. The AES-C version takes between 10 and 20 min to be completed [32].

The AES-C has been employed in three randomised controlled trials (RCTs) as a primary outcome in those with apathy and AD [33,34,35]. However, only the AES-C and AES-S versions were able to discriminate apathy from depression [32]. However, it is important to notice that different studies [36] confirmed good to excellent internal consistence of all the versions of the AES-C, with varying values for convergent validity and good discriminant validity. From the literature examined, it seems that the Apathy Evaluation Scale possesses these characteristics and can be validated on the AD population also in the Italian language. So, the aim of this study is to assess the specific reliability and validity of the Italian version of the AES-C scale to detect apathy both in amnestic MCI and mild AD patients. In the present paper, we therefore examined the psychometric properties and the invariance of the Italian Version of the AES-C in a sample of 107 amnestic MCI and AD patients compared with Age- and Sex-matched healthy controls.

## 2. Materials and Methods

### 2.1. Participants and Procedure

The study involved 214 participants (Table 1), divided into two groups. The experimental group consisted of 107 patients (34 males, 73 females) recruited from the U.O.S. Centro Alzheimer e Psicogeriatria, DSM ASP3, Catania, Italy where the new diagnostic criteria of the National Institute of Aging (NIA) and Alzheimer’s Association work group (2011) have been adopted both for AD and amnestic MCI patients. All patients selected for the present work were firstly recruited on their first access to the service, where they received the diagnosis for the first time. We tested 306 patients on their first access to the centre, but only 107 were included in the study based on the inclusion and exclusion criteria. The study was conducted in accordance with the Declaration of Helsinki, and the protocol was authorised by the Internal Ethics Review Board of the Department of Educational Sciences (Section of Psychology) of the University of Catania; research procedures followed all the indications provided by the guidelines of the AIP (Italian Association of Psychology) and its Ethical Council. The inclusion criteria included a diagnosis of MCI or probable Mild AD. As a screening for general cognitive impairment, we used the Italian standardised version of the Mini Mental State Examination (MMSE) [37,38], and included in the study patients with a total age and educational adjusted score ≥18 and ≤28. Patients with a recent history of cerebral ischaemia and/or a recent history of psychotic episodes were excluded. The control group was made up of 107 subjects (29 males, 78 females) of healthy volunteers, with MMSE scores ≥28. The neuropsychological evaluation involved the administration of various psychological tools. Questionnaires were administered individually: the scale was administered by the clinician to the patient without further interviews or input from the caregiver. Global cognitive function assessment has been carried out in MCI and AD patients by using the MMSE and Montreal Cognitive Assessment (MoCA) [39,40]. The Frontal Assessment Battery [41,42] was used to evaluate executive functions. Severity of depression was measured by means of the Italian version of the Hamilton Depression Rating Scale (HAMD-17) [43]. Apathy was assessed by the Apathy Evaluation Scale (AES-C). The HAM-D and AES-C were compiled by a clinician after a semi-structured interview with the patient to prevent depressed or apathetic subjects from being unable to report their symptoms adequately. Psychometric assessments were performed by two psychologists with a long experience in neuropsychological assessment and in particular in the field of behavioural and psychological symptoms of dementia.

### 2.2. Measures

#### 2.2.1. Mini Mental State Examination

The Mini Mental State Examination [37,38] constitutes a rapid and sensitive test for quantifying residual cognitive abilities for the documentation of their modification over time as a result of neurodegeneration. It is made up of twelve items through which seven cognitive functions are explored: time orientation, spatial orientation, immediate memory, attention and calculation, recall memory, language, visual-constructive praxis. Its administration requires a variable time from 5 to 15 min. It is a less demanding test, but it allows us to have a measure of global cognitive function that can also be used with the progression of the disease.

#### 2.2.2. Montreal Cognitive Assessment

The Montreal Cognitive Assessment (MoCA) [39,40] evaluates different cognitive domains: attention and concentration, executive functions, memory, language, visual-constructive skills, abstraction, calculation and orientation. The test administration time is 10 min; the maximum possible score is 30 points; a score equal to or higher than 26 is considered normal.

#### 2.2.3. Frontal Assessment Battery

The FAB [41,42], or Frontal Assessment Battery, is a short bedside cognitive and behavioural battery to assess frontal lobe functions. It consists of 6 cognitive and behavioural subtests: conceptualisation, mental flexibility, motor programming, sensitivity to interference, inhibitory control and environmental autonomy. In Apollonio’s [42] correction, the cut-off score is 13.50/18. The Frontal Assessment Battery is easy to administer at the bedside and is sensitive to frontal lobe and executive dysfunction.

#### 2.2.4. Hamilton Psychiatric Rating Scale for Depression

The Hamilton psychiatric Rating scale for Depression, i.e., HDRS or HAM-D, is undoubtedly the best known and most used psychometric tool in the world to evaluate depression’s severity. In its original formulation [43], the HAM-D was composed of 17 items, brought to 21 in the subsequent version [44]; besides these, numerous other versions have circulated with more or less arbitrary variants, the best known of which is the 24-item one (the added items are the feeling of helplessness, the loss of hope and the feeling of uselessness). The most widely used version is probably the one published in the NIMH “ECDEU Assessment Manual” [45]. It investigates 21 different areas that are crucial for the assessment of the subject’s depressive state. Areas are: depressed mood, guilt, suicidal thoughts, initial insomnia, intermediate insomnia, prolonged insomnia, work and interests, slowing of thought and speech, agitation, psychic anxiety, somatic anxiety, gastrointestinal somatic symptoms, somatic symptoms, genital symptoms, hypochondria, introspection, weight loss, diurnal symptomatology variation, depersonalisation, paranoid symptomatology, obsessive symptomatology. Each of the 21 areas represents a single item of the scale, to each of which the examiner, during the interview, must assign a score ranging from 0 (absent) to 4 (severe), or from 0 to 3 (clearly present), depending on the items and the severity of the symptoms. The score thus obtained indicates a possible depression if it is between 10 and 15 points, mild depression if it is between 16 events 25 points, moderate depression if it is between 26 and 28 points and severe depression if it is greater than 28 points.

#### 2.2.5. Apathy Evaluation Scale

The Apathy Evaluation Scale (AES-C) is a 4-point Likert scale consisting of 18 items. It requires 10–20 min to administer depending on the subject’s abilities and the version used. In the Clinician Version, a categorisation of items is indicated in the right column: B = behavioural item; C = cognitive item; E = emotional item. Items are worded with positive or negative syntax (+ or −); most are positive. The rating of Self-evaluation (SE) and quantifiable (Q) items are denoted in the right column of the AES-C. Scores for the AES-C range from 18 to 72. The cut-off score is 39–41, depending on which version of the AES is used. A clinical correlation suggests that these cut-offs are probably slightly low. In this work, the AES-C was compiled by a clinician after a semi-structured interview with the patient in order to prevent depressed or apathetic subjects from being unable to report their symptoms adequately. A back-translation procedure has been used during the adaptation to the Italian language, following the recommendations made by Beaton et al. [46]. The procedure was as follows: translation and adaptation of the original scale from Portuguese to Italian, back translation, and review committee. A bilingual Italian-English interpreter translated the English version of the AES-C into Italian. So, it was then translated back to English by a bilingual psychologist with a doctoral degree. The differences that emerged from the comparison between the two versions were discussed and addressed by the research group. Revisions were made to the Italian translation of the AES-C. No substantial difference has been highlighted between the final Italian version and the original English one.

### 2.3. Data Analysis

Linear structural equations models [47] were calibrated to test the hypothesised model. Tests were completed in AMOS 26.0 [48] by applying the maximum likelihood (ML) method. A sequence of CFA analyses was carried out on the dataset, to establish the best factor model to fit the data. The models’ goodness of fit was evaluated using the Tucker Lewis Index (TLI), the Comparative Fit Index (CFI), the Root Mean Square Error of Approximation (RMSEA) and the Standardised Root Mean Square Residual (SRMR). Furthermore, χ² values and Δχ² values between the competing models are presented, but they are sensitive to sample size [49], so the Akaike Information Criterion (AIC) was also presented along with the Bayesian Information Criterion (BIC) (lower values indicate a better fit). ΔCFI was also used, with values not exceeding 0.01 indicating that the models are equivalent in terms of fit [50]. Moreover, we conducted a confirmatory factor analysis (CFA) to confirm the factor structure of the AES-C. Next, a series of multiple group CFA were run, in which different, and progressively more stringent, forms of measurement equivalence were tested [51,52]. By establishing whether factor loadings, intercepts and residual variances are equivalent in a factor model that measures a latent concept, we can assure that comparisons that are made on the latent variable are valid across groups or time [53]. Other well-known analytical tools such as correlations were also used, which were implemented by using SPSS 27.0.

## 3. Results

### 3.1. Confirmatory Factor Analysis

At first, a model with one second-order factor and three first-order factors (Model 1: second order = 1-factor, Apathy; first order: 3-factors, Behavioural, Cognitive and Emotion) was measured; the results of the fit shown in Table 2 revealed that the model was good (χ^2^(129) = 241.388, SRMR = 0.05, RMSEA = 0.06, CFI = 0.93, TLI = 0.92, AIC = 325.388, BIC = 466.759). Model 1 was then compared with a three-factor model (Model 2: first order = 3-factors, Behavioural, Cognitive and Emotional), composed of three first-order factors with co-variances between them where the lower-level variables are observed variables containing errors (χ^2^ (132) = 349.059, SRMR = 0.05, RMSEA = 0.09, CFI = 0.84, TLI = 0.81, AIC = 472.059, BIC = 603.332). The first model of the two showed the best fit to the data, based on fit indexes, the AIC and the delta Chi-square value (Δχ²M2− M1(3) = 107.67). Model 1 was then compared to a one-factor model (Model 3: all 18 items predicted by a single factor), in which all the items were predicted by a single factor (χ^2^(135) = 404.847, SRMR = 0.06, RMSEA = 0.10, CFI =0.83, TLI = 0.80, AIC = 476.847, BIC = 698.023), and it showed again the best fit to the data (Δχ²M3−M1(6) = 163.459). Moreover, all factor loadings were significant at *p* < 0.001. Fit indexes for the tested models are presented in Table 2.

Results showed that Model 1 is the one with the best goodness of fit compared to the Italian sample we analyzed. This means that the Italian version of the AES-C confirmed the same factorial structure of the original version by Marin and colleagues.

### 3.2. Descriptive Statistic Normality of Distribution and Factor Loading of Model 1

Next, in Table 3 we report the list of items, the overall means with the standard deviations and the means by gender, the normality of the distribution and the factor loading of model 1 considered the most parsimonious, confirming the factorial structure of the scale. Critical values that exceed +2.00 or that are smaller than −2.00 indicate statistically significant degrees of non-normality. Descriptive statistics in Table 3 show that the data were normally distributed, with good skewness and kurtosis values. The results confirm the goodness of the scale and the normality of the distribution.

### 3.3. Convergent and Discriminant Validity

The convergent and discriminant validity of the AES-C was tested using the measures of the MMSE, MoCA, FAB and Hamilton. Table 4 shows descriptive statistics and the correlation matrix for the study variables. The data are reported by distinguishing the results between two different groups: the control group and experimental group (MCI and AD patients). There is a high negative correlation in the experimental group between the AES-C and the FAB (rs = −0.21, *p* < 0.001) and the MMSE (rs = −0.04, *p* < 0.001), while there is a positive correlation between the AES-C and the HAM-D scores (rs = 0.58, *p* < 0.001) and the MoCA (rs = 0.46, *p* <.001). These data are in line with Marin’s first AES validation study, in which moderate but statistically significant correlations were found between the AES-C and the HAM-D (*r* = 0.39) and the AES-C and the Hamilton Rating Scale for Anxiety (*r* = 0.35), indicating adequate discriminant validity. This confirms the fact that apathy, in addition to being considered as a symptom dimension of depression, presents itself as a construct connected to a psychopathological core but different from depression. In the control group, we found a high positive correlation between the AES-C and the HAM-D (rs = 0.22, *p* < 0.001) and the MoCA (rs = 0.17, *p* < 0.001), and a negative correlation between the AES-C and the FAB (rs = −0.22, *p* < 0.05). This latter result reflects the nature of the effects of frontal lobe dysfunction on the emotional aspects of evaluated subjects. There is in fact an anatomical correlation and a neurobiological link between frontal injury and the appearance of apathy [54,55]. Consequently, the negative correlation between the AES-C and the FAB suggests that in the absence of frontal lesions, the levels of apathy in healthy subjects are less elevated. The positive correlation between the AES-C and the MoCA in the control group was not expected. We hypothesize that this data could be linked to the presence of apathetic symptoms also in the healthy control group, where subjects were selected exclusively on the basis of their normal cognitive function (MMSE score ≥ 28). In fact, high apathy scores are compatible with a high score on the MoCA (general cognitive efficiency) rather than on the FAB, which measures frontal functions only. 

### 3.4. Multiple-Group Confirmatory Factor Analysis (MCFA)

Cross-validation comparisons were conducted by running a series of multiple-group CFA, in which different, and progressively more stringent, forms of measurement equivalence were tested [51]. We considered six models (configural, metric, scalar, error, variance and covariance) to test for measurement invariance across gender and type of group. The first multiple-group analysis tested a model of configural invariance (Model 1) by simultaneously evaluating the fit of male and female samples. The fit indices (χ^2^(258) = 421.58, *p* < 0.001; CFI = 0.91; SRMR = 0.043; RMSEA = 0.061) indicated a good fit for this model, supporting an equivalent solution made of one second-order factor with three first-order factors for the AES-C in the Italian context in Table 5. The fit of this configural model provides the baseline value against which all subsequently specified equivalence models are compared [56].

Model 2 was tested for metric invariance; Δχ^2^M2−M1(17) = 27.94 and ΔCFI = 0.001 suggested that Model 2 could be considered equivalent to Model 1 (Table 5). Thus, metric invariance was supported. 

Moreover, measurement scalar invariance (as tested by Model 3) and error invariance (Model 4) were found (Δχ^2^M3−M2(3) = 4.22, ΔCFI = 0.000; Δχ^2^M4−M3(22) = 69.84, ΔCFI = 0.002. 

The equivalence in factor variances was tested (Model 5), and it was found to be tenable (Δχ^2^M5−M4(15) = 29.53, ΔCFI = 0.001). Finally, the equivalence in factor covariances was tested (Model 6) by nesting the respective model with Model 5, and the result was that it was supported (Δχ^2^M6−M5(7) = 12.91, ΔCFI = 0.001). 

A second multigroup tested a model of configural invariance (Model 1) by simultaneously evaluating the fit of the experimental group and control group. The fit indices (χ^2^(96) = 452.91, *p* < 0.001; CFI = 0.90; SRMR = 0.054; RMSEA = 0.062) indicated a good fit for this model, supporting an equivalent solution made of one second-order factor with three first-order factors for the AES-C in the data sets for the experimental group and control group (Table 5). 

Model 2 was tested for metric invariance (Table 6). More importantly, Δχ^2^M1−M2(17) = 13.92 and ΔCFI = 0.000 suggested that Model 2 could be considered equivalent to Model 1. So, metric invariance was supported. 

The equivalence of the model is present also for error invariance (as tested by Model 3) and scalar invariance (Model 4) (Δχ^2^M2−M3(18) = 14.73, ΔCFI = 0.000; Δχ^2^M3−M4(11) = 19.6, ΔCFI = 0.000. 

Finally, the equivalence in factor variances (Model 5) and in factor covariances (Model 6) was tested, and the results were that it was supported (Δχ^2^M5−M4(15) = 22.03, ΔCFI = 0.000); (Δχ^2^M6−M5(8) = 37.12, ΔCFI = 0.000).

Results were totally satisfactory as the model fit proved to be invariant across both populations (Table 6). 

## 4. Discussion

Starting from the evidence that apathy can represent both a prodromal symptom and a risk factor for AD, in the present manuscript we analysed the psychometrics properties and invariance of the Italian Version of the Apathy Evaluation Scale—Clinician Version in a sample of 107 Italian MCI and AD patients. For this reason, different procedures were used, and different analyses were carried out. Confirmatory factory analysis confirmed that the AES-C Italian Version presents the same stability of one second-order factor and three first-order factors identified in Marin’s original study. Moreover, as in the original study, all items are predicted by a single general factor. So, in general, we can affirm that our results confirm the goodness of the scale. In 2007, Clarke and colleagues [57] conducted a review study of publications between January 1983 and December 2004 about AES-C psychometric properties. The authors provided detailed information about both reliability and validity, concluding that the scale has good psychometric properties, as firstly demonstrated by the original study by Marin et al. The original study [32] reported high internal consistency, with Cronbach’s alpha (α) = 0.90. This result was confirmed by subsequent studies where α exceeded 0.8, indicating that the scale is homogeneous. Marin et al. [32] also reported a good test-retest reliability, with a coefficient of 0.88 and a good inter-rater reliability (ICC = 0.94). Besides, there is strong evidence for good validity of the scale [57,58,59]. Content validity is supported by the judgement of various experts and by the fact that the AES-C has been used in many studies and across several health conditions. In different studies, convergent validity was assessed by comparing results of other measures of apathy, namely the AES-C self-rated, AES-C informant-rated and Apathy subscale of the Neuropsychiatric Inventory (NPI) [32,60,61]. Moreover, statistically significant correlations were observed between the AES-C and negative symptoms of the HAM-D, specifically, psychomotor retardation, lack of energy and insight and diminished work/interest. A similar result regards the correlations between the AES-C and reduced initiative, lack of emotional responsivity and inattention, as measured by the Montgomery and Asberg Depression Rating Scale (MADRS), as well as with the emotional withdrawal item of the Positive and Negative Syndrome Scale [62,63].

Our data agree with these findings and confirm the validity of the AES as a clinically relevant psychometric tool to detect apathy in MCI and mild AD patients. The convergent and discriminant validity of the AES-C was tested using the measures of cognitive impairment and the HDRS. In particular, it was planned to administer the Hamilton test, as in the original study, in order to evaluate also in the Italian version the ability of the AES to discriminate depression from apathy as a construct in and of itself. The results are in line with what was stated in the original article by Marin: in fact, the moderate but significant correlation between the AES-C and HAM-D confirms that apathy can not only be considered as a symptom of depression, but that it should be considered as a separate syndrome for which to intervene selectively to improve the quality of life of the subjects. Several previous studies showed the difference between the construct of apathy and that of depression. Already in 1998, Levy et al. [64] carried out research on a sample of subjects suffering from different neuropsychiatric diseases, showing that there was no correlation between apathy and depression and therefore confirming the separation of the two constructs, but they also found that apathy was strongly correlated with a low cognitive level measured by the Mini Mental State Examination. Successively, Starkstein et al. [65] have suggested that apathy is a behavioural dimension independent of depression. This seems very important considering that recent studies have shown that the higher the level of apathy, the more it is a predictor of the transition to dementia [2]; apathy alone or both apathy and depression are major risks to develop AD in MCI patients when compared to those with no NPS, as reported by Ruthirakuhan et al. [19]. Most of the epidemiological studies on apathy in the AD context refer to studies on the prevalence or change in apathy symptoms over time [66]. Apathy is predominant both in MCI and dementia. This prevalence is positively related to the severity of dementia [2]. A 3-year observational study of 332 MCI subjects showed a higher incidence of dementia in subjects with apathy ((HR) = 1.62) [67]. Moreover, an investigation using the Apathy Evaluation Scale (AES) in elderly individuals with normal cognition and MCI showed that apathy progression over time predicted the transition from MCI to dementia [68]. A very recent study on the predictivity of apathy for the transition to dementia was carried out by Roberto et al. [20], who identified and explored the predictivity of the conversion to dementia of four NPS profiles, distinguishing apathy from depression and demonstrating that apathy was a predictor of the conversion to dementia, as opposed to depression. Other studies have examined the discriminant validity by comparing AES-C scores and measures of depression and anxiety. Significant correlations were found between the AES-C and Hamilton Rating Scale for Anxiety; HAM-D scores of depressed mood, guilt or hopelessness, suicide and vegetative symptoms of depression; depressed mood symptoms on the MADRS; the Calgary Depression Scale for Schizophrenia (CDSS) and the NPI depression subscale. Other results that gave support to the construct validity are the significant negative correlations between the AES-C and physical arousal measures such as heart rate reactivity, diastolic blood pressure and mean arterial pressure [68].

Our study also examined for the first time the psychometric link between apathy and executive function. Specifically, when considering the healthy control group, interestingly we found a negative correlation between the AES-C and FAB. This result suggests that apathy is connected to damage of the prefrontal cortex and other brain structures [55]. In healthy subjects without brain damage, therefore, the score on the apathy test correlates negatively with that of the FAB, demonstrating the presence of a link between apathy and brain injury. Multiple-Group Confirmatory Factor Analysis (MCFA) was performed for measurement invariance across gender and type of group. Results showed that the same factor solution was invariant across gender (men, women), and they were totally satisfactory as the model fit proved to be invariant across both populations.

When moving to MCI and mild AD patients, it is well known that the availability of a valid and reliable psychometric tool for the measurement of apathy appears to be fundamental for evaluating and planning the treatment of apathy syndrome in the AD population, and in particular in an early phase of AD pathogenesis, such as amnestic MCI. A recent study demonstrate that the AES-C sub-scale can predict the progression from MCI to AD dementia [69] Other studies have evaluated the AES-C in mild and moderate AD patients. Radakovic et al. [70] examined the correlation between apathy and AD in a sample of 102 AD patients and 55 healthy controls. The aim of the study was to determine the validity and reliability of a multidimensional apathy measure, the Dimensional Apathy Scale (DAS) compared to the Apathy Evaluation Scale (AES), Geriatric Depression Short form (GDS-15), and Lawton Instrumental Activities of Daily Living (LIADL). The authors found in both subsamples a positive significant correlation between the DAS total score and AES (*r* = 0.75; *p* < 0.001), showing that apathy profiles in AD are heterogeneous, with additional specific impairments relating to awareness dependent on the apathy subtype [70]. The AES is a unidimensional measure, whereas apathy is established as a multidimensional construct. When considering the diagnostic criteria of apathy and in particular diminished motivation, reduced goal-directed cognitive activity and functional impairments, other psychometric tools such as the DAS should be used in combination with the AES in future clinical studies in early AD patients to evaluate the various dimensions of apathy better as well as additional specific impairments and specific clinical phenotypes such as the Executive-Initiation apathy detectable with the DAS eventually in combination with the AES-C.

Starting from the evidence of the key role of apathy as a risk factor for AD and the future development of disease-modifying strategies in amnestic MCI patients, the availability of a validated Italian version of the AES-C represents an essential step to plan future clinical studies in this field. Previous studies have found that the AES-S is a psychometrically sound measurement tool for assessing levels of apathy in a cognitively healthy middle-aged cohort at risk for AD [70], but our study demonstrates for the first time that the AES-C can be a useful tool to detect both apathy and executive dysfunction in amnestic MCI patients. 

Despite the goodness of the results obtained, the study has some limitations. One of these concerns the sample. Although the presence of a higher number of women than men confirms the literature, according to which AD disease is more frequent in the female gender, a more homogeneous sample between men and women would perhaps have been more adequate to represent a sample of the AD and not. Another limitation concerns the influence of the pharmacological interventions to which the subjects of the study could be subjected (such as antidepressants or cholinesterase inhibitors). This limitation is not present on our study, because no patients with a current depressive episode or treated with antidepressants were recruited in the study. Furthermore, we recruited only AD patients that did not receive a treatment with cholinesterase inhibitors, a drug class which might influence apathy scores. This might be considered as a limitation of our study, but it also helped us to exclude the impact of cholinesterase inhibitors on apathy scores in our AD sample. Further studies are needed to understand the impact of cholinesterase inhibitors on AES scores. A mixed sample of MCI and AD subjects all belonging to the same reference centre was used. Future studies could investigate the difference in the perception and presence of apathy in subjects undergoing treatment versus those not undergoing treatment, also to assess the impact of pharmacological vs. non-pharmacological treatments better. Finally, our control subjects showed high apathy scores. We cannot exclude the possibility that the presence of apathy in the control group may be related to the SARS-CoV-2 pandemic period during which the AES was administered.

## 5. Conclusions

From the results of the psychometric analyses conducted, it is possible to affirm the validity of the Italian version of the AES-C for the assessment of apathy both in MCI and in AD patients. Despite the limitations, our study leads to a real advance for future research and interventions. Considering the study and early identification of apathy in Alzheimer’s patients can be of fundamental importance to intervene promptly and improve the quality of life of MCI patients in an early phase of AD pathogenesis. This study allowed us to show that the AES-C shows good discriminating, convergent, criterion and construct values, excellent wearability and good reliability, and presents itself as an adequate tool in the Italian context to assess apathy both in MCI and AD subjects. 

## Figures and Tables

**Table 1:** Demographic and clinical characteristics of the sample.

Group	Gender	No.	Age (Mean ± DS)	Mild AD (No.)	MCI (No.)
**Experimental**					
	Male	34	75.62 ± 5.736	12	22
	Female	73	76.03 ± 7.297	19	54
	**Tot.**	107	75.90 ± 6.82	31	76
**Control**					
	Male	29	75.00 ± 6.984	-	-
	Female	78	73.70 ± 6.841	-	-
	**Tot.**	107	74.1 ± 6.87	-	-

**Table 2:** Fit indexes for models tested in CFA (Study 1).

	χ^2^	df	SRMR	RMSEA	RMSEA 90%-C.I.	CFI	TLI	AIC	BIC
Model 1 ^a^	241.388 *	129	0.05	0.064	0.051–0.076	0.93	0.92	325.388	466.759
Model 2 ^b^	349.059 *	132	0.05	0.096	0.086–0.108	0.84	0.81	472.059	603.332
Model 3 ^c^	404.847 *	135	0.06	0.10	0.089–0.112	0.83	0.80	476.847	698.023

*Note*. ^a^ Model 1: one second-order factor and three first-order factors. ^b^ Model 2: three first-order factors with covariances among them. ^c^ Model 3: all the items were predicted by a single factor. *: *p <* 0.001.

**Table 3:** Descriptive statistics (mean (M), standard deviation (SD), skewness, kurtosis) and factor loading of model 1.

	M	SD	Female (151)		Male (63)		Skewness	Kurtosis	Factor Loading Model 1
			M	SD	M	SD			
1. S/he is interested in things.	1.90	0.89	1.86	0.91	1.91	0.89	0.80	−0.07	0.905
2. S/he gets things done during the day.	1.66	0.81	1.54	0.78	1.71	0.82	1.13	0.68	0.950
3. Getting things started on his/her own is important to him/her.	1.92	0.99	1.81	0.99	1.96	0.99	0.78	−0.56	0.500
4. S/he is interested in having new experiences.	2.45	1.14	2.56	1.10	2.41	1.16	0.03	−1.42	0.532
5. S/he is interested in learning new things.	2.39	1.19	2.41	1.17	2.38	1.20	0.10	−1.51	0.549
6. S/he puts little effort into anything.	1.85	0.99	1.79	0.94	1.87	1.02	0.85	−0.48	0.691
7. S/he approaches life with intensity.	2.00	0.98	1.87	0.94	2.05	1.02	0.69	−0.61	0.613
8. Seeing a job through to the end is important to her/him.	1.45	0.70	1.30	0.56	1.51	0.75	1.50	1.66	0.719
9. S/he spends time doing things that interest her/him.	1.84	0.88	1.89	0.83	1.82	0.92	0.74	−0.36	0.810
10. Someone has to tell her/him what to do each day.	1.71	0.94	1.63	0.88	1.74	0.96	1.13	0.22	0.797
11. S/he is less concerned about her/his problems than s/he should be.	1.99	0.96	2.08	0.94	1.95	0.96	0.55	−0.78	0.674
12. S/he has friends.	1.79	0.96	1.67	0.86	1.83	1.00	1.07	0.13	0.901
13. Getting together with friends is important to her/him.	1.62	0.82	1.54	0.74	1.66	0.85	1.16	0.56	0.740
14. When something good happens, s/he gets excited.	1.43	0.714	1.46	0.69	1.42	0.72	1.56	1.68	0.749
15. S/he has an accurate understanding of her/his problems.	1.72	0.78	1.89	0.90	1.65	0.71	0.90	0.32	0.630
16. Getting things done during the day is important to her/him.	1.53	0.76	1.65	0.88	1.48	0.70	1.29	0.85	0.665
17. S/he has initiative.	1.92	1.003	1.98	1.04	1.89	0.99	0.73	−0.66	0.810
18. S/he has motivation.	1.95	0.99	1.98	0.99	1.93	0.98	0.71	−0.62	0.797

**Table 4:** Descriptive statistics and inter-correlations (N = 214) ^a^.

	Control Group		Experimental Group		α	1	2	3	4	5
	M	SD	M	SD						
1. MMSE	29.20	1.15	23.50	3.27	0.89	-	−0.10	0.09	0.11	−0.16 **
2. MoCA	28.53	1.57	18.33	4.38	0.77	0.63 **	-	−0.11	0.02	−0.17 **
3. FAB	16.24	1.33	11.31	3.28	0.87	0.52 **	0.55 **	-	−0.09	−0.22 **
4. Hamilton	4.37	2.58	10.29	7.35	0.76	0.14	0.08	−0.10	-	0.22 **
5. AES-C	28.35	7.8	37.58	11.0	0.91	−0.04	0.46 **	−0.21 **	0.58 **	-

Notes: MMSE = Mini Mental State Examination; MoCA = Montreal Cognitive Assessment; FAB = Frontal Assessment Battery; Hamilton = Hamilton psychiatric Rating scales For Depression; AES-C = Apathy Evaluation Scale—Clinician Version; *p* scores: * < 0.05, ** < 0.001. ^a^ Correlations among experimental data are below the diagonal; correlations among control group data are above the diagonal.

**Table 5:** Fit statistics for measurement invariance by gender.

Model	χ^2^(df)	CFI	SRMR	RMSEA (90% CI) *p* Close	ΔCFI
1. Configural Invariance	421.58 (258)	0.91	0.04	0.06 (0.045–0.064)	-
2. Metric Invariance	449.52 (275)	0.90	0.04	0.06 (0.045–0.064)	0.001
3. Scalar Invariance	453.74 (278)	0.90	0.04	0.06 (0.045–0.064)	0.000
4. Measurement error Invariance	523.58 (300)	0.90	0.04	0.06 (0.045–0.064)	0.002
5. Structural Variance Invariance	553.11 (315)	0.90	0.04	0.06 (0.045–0.064)	0.001
6. Structural Covariance Invariance	566.02 (322)	0.90	0.04	0.06 (0.045–0.064)	0.001

**Table 6:** Fit statistics for measurement invariance by experimental group and control group.

Model	χ^2^(df)	CFI	SRMR	RMSEA (90% CI) *p* Close	ΔCFI
1. Configural Invariance	452.91 (256)	0.90	0.05	0.06 (0.051–0.069)	-
2. Metric Invariance	466.83 (273)	0.90	0.05	0.06 (0.054–0.080)	0.000
3. Scalar Invariance	481.56 (291)	0.90	0.05	0.06 (0.054–0.080)	0.000
4. Measurement error Invariance	501.16 (302)	0.90	0.05	0.06 (0.054–0.080)	0.000
5. Structural Variance Invariance	523.19 (317)	0.90	0.06	0.06 (0.054–0.080)	0.000
6. Structural Covariance Invariance	560.31 (325)	0.90	0.06	0.06 (0.054–0.080)	0.000

## Data Availability

The data presented in this study are available on request. The data are not publicly available due to privacy restrictions.
